# Incidence of aspiration pneumonia during hospitalization in Japanese hospitalized cases did not increase whereas concern factors were exacerbated in a time-dependent manner: analysis of Diagnosis Procedure Combination (DPC) data

**DOI:** 10.3164/jcbn.17-119

**Published:** 2018-04-11

**Authors:** Sayuri Nonaka, Susumu Fujii, Megumi Hara, Shigeki Morita, Eisaburo Sueoka, Koichi Node, Kazuma Fujimoto

**Affiliations:** 1 Saga University Hospital, 5-1-1 Nabeshima Saga, Saga 849-8501, Japan; 2 Department of Preventive Medicine, Saga University, 5-1-1 Nabeshima Saga, Saga 849-8501, Japan

**Keywords:** activities of daily livingaspiration pneumonia, age concern factors, diagnosis procedure combination/per-diem payment system, dysphagia, hospitalized cases, dysphagia, male

## Abstract

This study aimed i) to investigate about items with high relevance for aspiration pneumonia during hospitalization among cases evaluated using Diagnosis Procedure Combination data, and ii) to determine whether the concern factors for aspiration pneumonia during hospitalization were exacerbated with the trend of the time. The Diagnosis Procedure Combination data were gathered from 2010 through to 2015 with 63,390 cases at Saga University Hospital. The occurrence of concern factors of aspiration pneumonia during hospitalization were compared in the two time periods set (2010–2012 and 2013–2015). The concern factors included: male, age, dysphagia at admission and during hospitalization, use and days in the emergency care unit or high care unit, use of the intensive care unit, and use of an ambulance. Age, dysphagia, and use of the intensive care unit were time-dependently exacerbated. The incidence of aspiration pneumonia during hospitalization in hospitalized cases did not differ between years 2010–2012 and 2013–2015. Aspiration pneumonia during hospitalization complicated with surgery and number days in the emergency care unit or high care unit diminished in years 2013–2015. Despite an increased concern of aspiration pneumonia during hospitalization, the complication rate of aspiration pneumonia during hospitalization did not increase.

## Introduction

Pneumonia was the third most common disease among causes of mortality in Japan in 2014.^(1)^ Several Japanese studies indicate that pneumonia in the elderly (aged ≥70 years) during hospitalization is caused by aspiration, and that aspiration pneumonia is one of the main causes of mortality in the elderly.^(2,3)^ This is of utmost concern because the aged population in Japan will increase dramatically in coming years, comprising 30% (36,000,000) of the country’s entire population in 2025.^(4)^

In Japan, use of a Diagnosis Procedure Combination/per-diem payment system (DPC/PDPS) for treating hospitalized patients receiving acute care began in April 2003.^(5–9)^ The system was introduced into 1,667 general acute care hospitals (495,000 beds) in 2016.^(10)^ The DPC data are explained briefly as follows. Anonymized DPC data include a file 1 form that records inpatients’ medical histories, E·F files that detail medical care/treatment received (procedure, material, and medication), and a D file that outlines comprehensive points, service-based points, and DPC codes. The file 1 form consists of major classification items such as “data attributes”, “admission/discharge information”, “diagnostic information”, “surgery information”, “treatment information”, and “re-hospitalization information”. The above items are major classification items of the 2010 version (from April 2010 to March 2011) and are revised once every two years.^(11)^ The “diagnostic information” category encompasses “name of main injury/disease name”, “name of injury/disease as a cause of hospitalization”, “name of injury/disease with the largest number of medical resources”, “name of injury/disease with the second largest number of medical resources”, “name of comorbidity at the time of hospitalization with maximum of 4 diseases”, and “name of disease occurred during hospitalization with maximum of 4 diseases”.^(12)^

The present study aimed to i) investigate about items with high relevance for aspiration pneumonia during hospitalization among cases evaluated using DPC data, and ii) determine whether concern factors for aspiration pneumonia in the hospitalized cases changed in a time-dependent manner.

## Materials and Methods

Saga University Hospital’s DPC data were examined and 63,390 discharged cases from January 2010 through to December 2015 were included in the study. The cases were classified into two groups: aspiration pneumonia developed during hospitalization (group I), and non-aspiration pneumonia developed during hospitalization (group II). Aspiration pneumonia cases were defined according to the description of “J690: aspiration pneumonia” in the International Statistical Classification of Diseases and Related Health Problems, Tenth Revision (ICD-10)^(13–15)^ and based on DPC data, particularly “diagnostic information” of the major classification items in the file 1 form. Cases which developed aspiration pneumonia upon admission were excluded. The study evaluated and drew relationships using DPC data for groups I and II, and concern factors for onset of aspiration pneumonia were determined. Concern factors in this study are almost all items that can be obtained from DPC data.

The evaluated concern factors were male, age, surgery, number of days spent hospitalized before surgery, number of days spent hospitalized after surgery, total number of days spent in hospital, number of days spent in general ward, use of emergency care unit or high care unit (ECU/HCU), number of days in ECU/HCU, use of intensive care unit (ICU), number of days in ICU, emergency hospitalization, use of an ambulance, dysphagia at admission, and dysphagia during hospitalization.^(11)^ These factors were obtained from file 1 form, but number of days spent in general ward, use of emergency care unit or high care unit (ECU/HCU), number of days in ECU/HCU, use of intensive care unit (ICU), and number of days in ICU were obtained from E and F files. Dysphagia patients were defined according to ICD-10 “R13: dysphagia” and based on “name of comorbidity (maximum 4 diseases) at the time of hospitalization” and “name of disease (maximum 4 diseases) occurred during hospitalization”. Of concern factors of file 1 form, factors that differ depending on the revised year and factors that are not essential were excluded.

All cases were also classified according to admission period: January 2010–December 2012 (group A), and January 2013–December 2015 (group B). Thus, whether or not concern factors were increased in a time-dependent manner was examined. Participants were further classified into group A with aspiration pneumonia during hospitalization (group A-I) and group B with aspiration pneumonia during hospitalization (group B-I). The incidence rate of actual aspiration pneumonia during hospitalization and each of these group’s concern factor incidence rates were calculated. *T* test or Mann-Whitney *U* test was applied for continuous variables, and chi-square test was applied for categorical variables. The ORs of factors were analyzed with logistic regression using Stat Flex (Windows) ver. 6 software. Statistical significance was defined as *p*<0.05.

## Results

Of 63,390 cases, group I and group II comprised 426 and 62,964 cases, respectively. Table 1 shows the concern factors for aspiration pneumonia during hospitalization (group I) evaluated using univariate analysis, which included: male, age, number of days spent hospitalized after surgery, number of days spent in the hospital and the general ward, use of the ECU/HCU, number of days in the ECU/HCU, use of the ICU, emergency hospitalization, use of an ambulance, dysphagia at admission, and dysphagia during hospitalization. More cases in group II than group I underwent surgery. Table 2 shows the concern factors for aspiration pneumonia during hospitalization in univariate analysis as evaluated by multivariate analysis, which indicates that most concern factors, except for emergency hospitalization, were increased for aspiration pneumonia during hospitalization.

Table 3 shows the concern factor changes for aspiration pneumonia during hospitalization for hospitalized cases at Saga University Hospital between two periods: January 2010–December 2012 (*n* = 31,443, group A) and January 2013–December 2015 (*n* = 31,947, group B). The mortality rate did not differ in the two groups (group A: 1.83%, 574 cases; group B: 1.75%, 558 cases). Age, use of the ICU, and dysphagia at admission and during hospitalization were more common in group B, whereas number of days in the general ward, use of the ECH/HCU, and number of days in the ECU/HCU was higher in group A.

Table 4 compares the incidence rate of actual aspiration pneumonia during hospitalization and the concern factors among the aspiration pneumonia during hospitalization cases between the two study periods: January 2010–December 2012 (*n* = 229) and January 2013–December 2015 (*n* = 197). Regarding the incidence rate of aspiration pneumonia during hospitalization, there was no statistical significance between the two periods, but improved in a time-dependent manner (229/31,443: 0.73% vs 197/31,947: 0.62%). Aspiration pneumonia during hospitalization was complicated in cases which underwent surgery and the number days spent in the ECU/HCU improved (i.e., decreased) in a time-dependent manner.

## Discussion

The present study showed that concern factors of aspiration pneumonia during hospitalization in DPC data were male, age, dysphagia at admission and dysphagia during hospitalization, use of the ECU/HCU, number of days in the ECU/HCU, use of the ICU, use of an ambulance, and number of days in the general ward. Vital statistics in Japan (2016 edition) demonstrated that pneumonia mortality rate was higher in males than in females.^(1)^ Another study showed that prevalence of aspiration pneumonia increased with aging and most patients with pneumonia aged ≥70 years suffered from aspiration pneumonia.^(2)^ The committee for the Japanese Respiratory Society guidelines for management of respiratory infections showed aspiration pneumonia was complicated in dysphagia,^(16)^ and several additional studies indicated that dysphagia was a major cause of aspiration pneumonia.^(17–20)^ These data were consistent with our results that dysphagia at admission and during hospitalization were the main concern factors for aspiration pneumonia during hospitalization. The concern factors of aspiration pneumonia during hospitalization indicated in the present study, including use of the ECU/HCU, number of days in the ECU/HCU, use of the ICU, and use of an ambulance showed that these cases were serious cases. These were consistent with the study of the onset influence factors of infections preceding hospitalization. Also, I thought that prolonging the number of hospital days is not an influential factor but should be considered as an extension required for treatment.^(21)^

Concern factors for aspiration pneumonia during hospitalization were exacerbated in a time-dependent manner. Namely, ratio of aged cases, use of the ICU, and dysphagia at admission and during hospitalization increased in years 2013–2015 compared with years 2010–2012. Additionally, the complication rate of aspiration pneumonia during hospitalization in the cases which received the surgical operation in years 2013–2015 improved time-dependently.

The present study had several limitations. First, the DPC data could neither estimate the timing in complications of aspiration pneumonia during hospitalization nor perform a follow-up survey of the disease. Second, severity of the illness and impaired activities of daily living were speculated by use of the ECU/HCU or the ICU, and use of an ambulance, but not by examination of patients’ actual conditions. Third, the relationship between prescribed medicines and aspiration pneumonia was not detected by the DPC data, because i) the prescription data before hospitalization were not detected from the DPC data and ii) the relationship between the onset day of aspiration pneumonia and the prescription period during hospitalization was not strictly identified. Prescription trend was excluded from the present study, although several medicines including proton pump inhibitors and statins were risks for pneumonia^(22–25)^ which warrant further exploration for the DPC data analysis.

The findings of this study suggest that the concern of aspiration pneumonia during hospitalization is exacerbated over time, concomitant with an increase in concern factors. Additionally, the findings suggest the possibility of suppressing the onset by carrying out preventive measures on cases which are susceptible to aspiration pneumonia during hospitalization.

## Authors’ Contributions

Principal investigator, data collection, statistical analysis, and manuscript preparation: Sayuri Nonaka. Statistical analysis: Megumi Hara. Data collection and statistical analysis: Susumu Fujii. Data collection: Shigeki Morita, Eizaburo Sueoka, Koichi Node, and Kazuma Fujimoto.

## Figures and Tables

**Table 1:** Concern factors for aspiration pneumonia evaluated by chi square test or Mann-Whitney *U* test

	Group I: aspiration pneumonia group (*n* = 426)	Group II: non-aspiration pneumonia group (*n* = 62,964)	*p* value
Male	268 (62.9%)	32,335 (51.4%)	<0.001*
Age	73.6 ± 14.9	56.7 ± 24.5	<0.001
With surgery	193 (45.3%)	34,813 (55.3%)	<0.001*
Number of days hospitalized before surgery	7.2 ± 13.6 (*n* = 193)	3.4 ± 7.2 (*n* = 34,813)	0.169
Number of days hospitalized after surgery	30.3 ± 33.4 (*n* = 193)	13.2 ± 19.7 (*n* = 34,813)	<0.001
Number of total days in hospital^†^	31.8 ± 30.0	16.6 ± 21.5	<0.001
Number of days in the general ward	27.4 ± 30.2 (*n* = 409)	16.1 ± 21.1 (*n* = 62,563)	<0.001
Use of ECU/HCU	243 (57.0%)	5,631 (8.9%)	<0.001*
Number of days in ECU/HCU	8.8 ± 4.8 (*n* = 243)	4.7 ± 4.4 (*n* = 5,631)	<0.001
Use of ICU	36 (8.5%)	2,948 (4.7%)	<0.001*
Number of days in ICU	5.1 ± 4.4 (*n* = 36)	5.1 ± 6.6 (*n* = 2,948)	0.273
Emergency hospitalization	300 (70.4%)	18,602 (29.5%)	<0.001*
Use of an ambulance	273 (64.1%)	9,217 (14.6%)	<0.001*
Dysphagia			
at admission	25 (5.9%)	251 (0.4%)	<0.001*
during hospitalization	53 (12.4%)	451 (0.7%)	<0.001*
Mortality	58 (13.6%)	1,074 (1.7%)	<0.001*

Data are mean ± SD. *Evaluated by chi square test. ^†^The sum of days in general ward, ECU/HCU, and ICU. ECU, emergency care unit; HCU, high care unit; ICU, intensive care unit.

**Table 2:** Concern factors for aspiration pneumonia evaluated by multivariate logistic regression analysis

Variable	β	SE (β)	*z* value	*p* value	OR	95% CI
Male	0.445	0.105	4.234	<0.001	1.56	1.270–1.917
Age	0.04	0.004	10.898	<0.001	1.041	1.033–1.048
With surgery	–0.370	0.11	3.38	0.001	0.69	0.557–0.856
Number of days in general ward	0.007	0.001	6.039	<0.001	1.007	1.005–1.010
Use of ECU/HCU	0.613	0.186	3.302	0.001	1.847	1.283–2.658
Number of days in ECU/HCU	0.124	0.013	9.635	<0.001	1.132	1.104–1.161
Use of ICU	0.835	0.202	4.135	<0.001	2.306	1.552–3.426
Emergency hospitalization	0.187	0.171	1.091	0.275	1.205	0.862–1.685
Use of an ambulance	0.85	0.177	4.813	<0.001	2.34	1.655–3.307
Dysphagia						
at admission	1.632	0.239	6.83	<0.001	5.115	3.202–8.171
during hospitalization	1.125	0.175	6.423	<0.001	3.081	2.185–4.342

Conformity degree index of the regression: AIC = 3,996.174, AUC = 0.876. OR, odds ratio; 95% CI, 95% confidence interval; β, regression coefficient; SE (β), standard error (β); ECU, emergency care unit; HCU, high care unit; ICU, intensive care unit.

**Table 3:** Analysis of whether concern factors for aspiration pneumonia increased in recent years by comparing groups A and B

	Group A: January 2010–December 2012 (*n* = 31,443)	Group B: January 2013–December 2015 (*n* = 31,947)	*p* value
Male	16,120 (51.3%)	16,483 (51.6%)	0.410*
Age	55.8 ± 25.0	57.8 ± 24.0	<0.001
With surgery	16,987 (54.0%)	18,019 (56.4%)	<0.001*
Number of days in general ward	16.7 ± 21.5 (*n* = 31,166)	15.6 ± 20.9 (*n* = 31,806)	<0.001
Use of ECU/HCU	3,092 (9.8%)	2,782 (8.7%)	<0.001*
Number of days in ECU/HCU	5.1 ± 4.3 (*n* = 3,092)	4.6 ± 4.8 (*n* = 2,782)	<0.001
Use of ICU	1,421 (4.5%)	1,563 (4.9%)	0.027*
Use of an ambulance	4,677 (14.9%)	4,813 (15.1%)	0.500*
Dysphagia			
at admission	115 (0.4%)	161 (0.5%)	0.008*
during hospitalization	120 (0.4%)	384 (1.2%)	<0.001*

Data are mean ± SD. *Evaluated by chi square test. ECU, emergency care unit; HCU, high care unit; ICU, intensive care unit.

**Table 4:** Analysis of concern factors by comparing aspiration pneumonia of groups A and B

	Group A-I: aspiration pneumonia group in group A (*n* = 229)	Group B-I: aspiration pneumonia group in group B (*n* = 197)	*p* value
Incidence rate	0.7%: 229/31,443	0.6%: 197/31,947	0.085^†^
Incidence in males	0.9%: 139/16,120	0.8%: 129/16,483	0.426^†^
Age	73.1 ± 14.7	74.1 ± 15.3	0.515
Incidence in cases with surgery	0.7%: 111/16,987	0.5%: 82/18,019	0.012^†^
Number of days in general ward	27.2 ± 33.1 (*n* = 215)	27.6 ± 26.8 (*n* = 194)	0.095
Incidence in cases using ECU/HCU	4.0%: 125/3,092	4.2%: 118/2,782	0.702^†^
Number of days in ECU/HCU	9.8 ± 4.1 (*n* = 125)	7.8 ± 5.3 (*n* = 118)	<0.001
Incidence in cases using ICU	1.3%: 18/1,421	1.2%: 18/1,563	0.774^†^
Incidence in cases using ambulance	3.0%: 139/4,677	2.8%: 134/4,813	0.584^†^
Incidence in cases with dysphagia			
at admission	7.8%: 9/115	9.9%: 16/161	0.547^†^
during hospitalization	13.3%: 16/120	9.6%: 37/384	0.249^†^

Data are mean ± SD.^†^Evaluated by chi square test. ECU, emergency care unit; HCU, high care unit; ICU, intensive care unit.

## Abbreviations

DPC: diagnosis procedure combination
ECU: emergency care unit
ICU: intensive care unit
